# Primary Dysmenorrhea and Menstrual Symptoms in Indian Female Students: Prevalence, Impact and Management

**DOI:** 10.5539/gjhs.v8n8p135

**Published:** 2015-12-17

**Authors:** Shabnam Omidvar, Fatemeh Bakouei, Fatemeh Nasiri Amiri, Khyrunnisa Begum

**Affiliations:** 1 Department of Midwifery, Babol University of Medical Sciences Babol, Iran; 2 Department of Studies in Food Science and Nutrition, University of Mysore, Mysore, India

**Keywords:** primary dysmenorrhea, impact, management, menstrual symptoms

## Abstract

**Background:**

Dysmenorrhea is the most common gynecological problem among females and it is defined as cramping pain in the lower abdomen occurring just before or during menstruation. Menstrual symptoms are a broad collection of affective and somatic concerns that occur around the time of menses. The effect and importance of dysmenorrhea is very wide, therefore managing the problem is important.

**Objective:**

To ascertain the prevalence, and impact of primary dysmenorrhea in student girls and their management behaviors.

**Methods and Materials:**

A Cross-sectional study was conducted on 1000 healthy females aged 11-28 years. Standardized Self-reporting questionnaires were used to obtain relevant data. Pain intensity was assessed by using the Numerical Pain Scale (NPS). Data was analyzed by SPSS version 16.

**Results:**

Prevalence of dysmenorrhea was 70.2%. Majority of the subjects experienced pain for one or 1-2 days during menstruation. 23.2% of the dysmenorrheic girls experienced pain for 2-3 days. The most common symptom in both dysmenorrheic and non dysmenorrheic girls during the menstrual periods was tiredness and second most prevalent symptom was back pain.

Females experiencing mild pain on an average absented for one and half day a month while 2.1±1.2 and 2.5±1.3 days for those who experienced moderate and severe forms of dysmenorrhea respectively. A small proportion of girls sought pharmacological management (25.5%) and 83.2% depended on non-pharmacological methods. Only 14.2% had sought medical advice.

**Conclusion:**

Sub optimal use of the medical advice and the barriers to seek medical attention by dysmenorrheic females need exploration. It is important that health education on puberty and menstruation is regarded as inadequate for many girls in India.

## 1. Introduction

Dysmenorrhea is a cyclical lower abdominal or pelvic pain which may also radiate to the back and thighs; it occurs before or during menstruation, or both (Raine-Fenning, 2005). Cramps and pain are experienced in the lower abdominal after regular ovulation is established. It begins soon after menarche (Reddish, 2006). It is the most common gynecologic complaint among adolescent and adult females (Doty & Attaran, 2006; Polat, Celik et al., 2009).

Menstruation-associated symptoms are a broad collection of affective and somatic concerns that occur around the time of menses (Harel, 2006; Negriff, 2009). Symptoms, such as headache, vomiting, tiredness, dizziness, and diarrhea are few commonly experienced menstrual symptoms. Symptoms typically start at the onset of menstrual flow or occur within a few hours before or after onset, and last for the first 24-48 hours.

The epidemiology of primary dysmenorrhea is difficult to establish since it is a symptom that is perceived differently by different women and also diversity in diagnostic criteria that is often used. The prevalence estimates range from 25 to 90% among women and adolescents (Okusanya, 2009). Studies from India reported the prevalence range between 50 to 87.8% (George & Bhaduri, 2002; Patel, 2006; Nair, 2007; Singh, 2008; Agarwal & Agarwal, 2010). Other studies reported that dysmenorrhea affects up to 90% of women of childbearing age to varying degrees (Weissman, 2004). Pain is significant in 5-20% who report severe dysmenorrhea or pain that prevents them from participating in their usual activities. In the United States, the annual economic loss has been estimated as 600 million work hours worth 2 billion dollars. The effect and importance of dysmenorrhea is very wide and not restricted to its socio-economic impact (Raine-Fenning, 2005).

Although etiology of dysmenorrhea is not fully understood but the cause of pain at, or around, the time of menses is believed to be due to the production of prostaglandins in the endometrium in an ovulatory cycle. There are some reports that indicate the levels of prostaglandin F2α measured in menstrual fluid from tampons and found to be twice higher in the dysmenorrheic as against the non dysmenorrheic women (Harel, 2006). As a remedial measure significant beneficial effect of prostaglandin inhibitors is reported in patients with dysmenorrhea. Despite the evidence supporting a link between prostaglandin levels and dysmenorrhea, it is important to recognize that the explanation for menstrual pain is not as simplistic as the cyclic production of one hormone. Women with dysmenorrhea may have complex alterations in hormonal patterns that exist throughout the cycle (Sharp, 2002). Treatment for dysmenorrhea is aimed at relieving symptoms and is best treated with analgesics that are prostaglandin inhibitors (Shilpa, 2008). The objective of this study was to ascertain the prevalence of dysmenorrhea and menstrual symptoms in Indian girls, its impact on their performance and treatment approaches.

## 2. Methods and Materials

The present study was of a Cross-sectional design conducted in an urban area in a major city in South India. The selected subjects were explained about the protocol and purpose of the study and were requested to complete the questionnaires to gather information relating to demographic feature, menstrual pain, intensity of pain, pain characteristics, menstrual symptoms, impact of dysmenorrhea and symptoms on their daily chores and their menstrual pain management methods. One thousand healthy young females aged 11-28 years formed the study population. A purposeful sampling was adopted to select unmarried girls; also those who volunteered to give complete and correct information were included for the study. The demographic information included family details relating to family size and type, chronological age and age at menarche. Possession of costly goods like vehicles, computer, TV, DVD, refrigerator, phones was also obtained and this information was used to compute Socio Economic Status (SES); it was designated as low, middle and high SES. The Numerical Pain Scale developed by McCaffery and Beebe (1994) was used for assessing pain intensity (McMaffery, 1994). The research protocol was approved by the Ethical Committee, University of Mysore.

The data was analyzed using SPSS for Windows version 16. Descriptive statistics was used to determine mean and percentages. The categorical data was analyzed using Chi-sq, correlation and regression analyses.

## 3. Results

Table 1 provides participants general and family information. The proportion of participants belonging to the four age categories (11-15, 16-18, 19-21, >22 years) were essentially similar. It is evident that majority of the selected girls belonged to Hindu families, while those from Islam, Christianity and other religions varied from 1.3-12.3%. Nearly half of the subjects had completed or presently studying in 10^th^ standard or lower than that, 33.2 and 19.4% were studying in pre university and graduate classes. It can be seen that regardless of the SES; the predominant family type was nuclear family with members ≤4. Joint family and the extended families together formed 14% wherein the size of the family varied from 5-8 members. Large families with more than 8 members were insignificant. More than half of the subjects belonged to medium SES followed by low SES (36.7%). It was surprising that a considerable percentage of the selected subjects mentioned to have a positive family history of menstrual pain.

**Table 1:** Demographic information about the participants

Information		N	%
**Age(years)**	11-15	226	22.6
	16-18	252	25.2
	19-21	271	27.1
	≥22	251	25.1
**Religion**	Hindu	836	84.0
	Muslim	123	12.3
	Christian	24	2.4
	Other	13	1.3
**Education**	Up to 10^th^std	469	47.0
	Pre university	332	33.2
	Graduation	194	19.4
	Post graduation	4	0.4
**Type of family**	Joint	64	6.4
	Nuclear	852	85.7
	Extended	78	7.8
**Family size**	≤4	538	53.9
	5-8	442	44.3
	>8	18	1.8
**SES**	Low	367	36.7
	Medium	529	52.9
	High	104	10.4
**Family history in menstrual pain**	Yes	622	64.5
	No	342	35.5

70.2% of the participants experienced menstrual pain, i.e. they were dysmenorrheic (Table 2). It was a point of concern since it is an indication of discomfort and poor work efficiency of the person. Length of pain varied for one day to the entire period of menstruation. Majority of them experienced pain for one or 1-2 days during menstruation. Furthermore, it is important to mention that 23.2% of the participants experienced pain for 2-3 days and a small percent had for the entire period.

**Table 2:** Dysmenorrhea and associated features: occurrence of dysmenorrhea among the selected females

Dysmenorrhea variables			N	(%)
**Menstrual pain**	Yes		698	70.2
	No		296	29.8
**Pain length**	One day		213	30.1
	1-2 days		288	40.7
	2-3 days		164	23.2
	Entire of period		42	6
**Pain intensity**	Mil		211	30.2
	Moderate		333	47.7
	Severe		154	22.1
**Menstrual symptoms**	Yes		909	91.3
	No		87	8.7
**Frequency of dysmenorrhea occurrence**	More frequently (every month)		458	68.3
	Less frequently (Once in 3 month)		134	20.0
	Rarely(Once in 6 months)		79	11.8
**Absenteeism because of menstrual pain/symptoms**	No		349	49.9
	Yes	Half day	97	13.9
		1 day	173	24.8
		2 days	44	6.3
		≥3 days	36	5.2
**Taking analgesics**	Yes		178	25.6
	No		517	74.4

It was of interest to investigate the frequency of occurrence of dysmenorrhea. Intra and inter individual variations have been reported in the frequency of dysmenorrhea. A variety of factors are indicated to influence dysmenorrheal, most importantly are the psychological factors like stress, nutritional factors and hormonal effects. In the study population, girls with dysmenorrhea, (68.3%) mentioned consistent pain during periods. On the other hand 20 and 11.8% dysmenorrheic girls reported having pain; less frequently and rarely respectively (Table 2). Approximately 50% of the girls in the study group remained absent from schools or colleges due to pain and/or discomfort related to menstruation. Only 25.6% of the dysmenorrheic participants mentioned to manage pain by taking analgesic Tablets.

Our results exhibited that 69.7% of subjects were moderately affected, limiting their working capacity and 7.6% of both dysmenorrheic and non dysmenorrheic mentioned to have a clear inhibition of their activities.

Table 4 presents association between certain subjective factors with intensity of menstrual pain. It is obvious that age and SES did not have any association with pain intensity. Religion exhibited small but significant association to pain; wherein higher percentage of girls from Hindu religion were found to have moderate pain followed by Christians while higher percentage of Muslim girls experienced severe pain.

**Table 3:** Ability of the participants to attend daily chores during menstrual time: differences between dysmenorrheic and non dysmenorrheic females

Attending daily chores	Dysmenorrheic	Non dysmenorrheic	Total	Chi Square
**Unaffected**	**155(22.7)**	**134(48.2)**	**289**	63.516^***^
**Moderately affected**	**477(69.7)**	**123(44.2)**	**600**	
**Clearly inhibited**	52(7.6)	21(7.6)	73	

**Table 4:** Association between subjective factors and pain intensity among dysmenorrheic girls

Variables		N (%)	Menstrual pain intensity			Chi sq

			Mild n (%)	Moderate n (%)	Severe n (%)	
**Age (Yrs)**	12-15	156(22.3)	48(22.7)	72(21.6)	36(23.4)	4.103^Ns^
	16-18	159(22.8)	42(19.9)	84(25.2)	33(21.4)	
	19-21	203(29.1)	70(33.2)	89(26.7)	44(28.6)	
	>21	180(25.8)	51(24.2)	88(26.4)	41(26.6)	
**Menarche age (Yrs)**	10-12	191	52(25.7)	93(28.7)	46(30.5)	1.95^Ns^
	13-14	387	117(57.9)	183(56.5)	87(57.6)	
	15-17	99	33(16.3)	48(14.8)	18(11.9)	
**SES**	Low	261(37.4)	81(38.4)	127(38.1)	53(34.4)	0.804^Ns^
	Middle	357(51.1)	107(50.7)	168(50.5)	82(53.2)	
	High	80(11.5)	23(10.9)	38(11.4)	19(12.3)	
Anemia* (before 6 months)	Yes	116(16.8)	27(12.9)	38(11.5)	51(33.8)	40.08^***^
	No	575(83.2)	183(87.1)	292(88.5)	100(66.2)	
**Exercise**	Yes	88(12.7)	33(15.7)	33(10.0)	22(14.3)	5.91^Ns^
	No	480(69.3)	137(65.2)	233(70.8)	110(71.4)	
	sometimes	125(18.0)	40(19)	63(19.1)	22(14.3)	
**Absenteeism**	None	330(49.3)	128(67.0)	151(46.3)	51(33.6)	67.03^***^
	Half day	90(13.5)	30(15.7)	45(13.8)	15(9.9)	
	1 day	170(25.4)	25(13.0)	90(27.6)	55(36.2)	
	2 days	44(6.6)	5(2.6)	25(7.7)	14(9.2)	
	3 days	35(5.2)	3(1.6)	15(4.6)	17(11.2)	
Working ability*	Unaffected	155	75(36.4)	65(19.8)	15(10.0)	49.28^***^
	Moderately affected	477	126(61.2)	238(72.6)	113(75.3)	
	Clearly inhibited	52	5(2.4)	25(7.6)	22(14.7)
**Percentage of absenteeism**			33.0	53.7	66.4
**Mean absent days**			1.6±0.9	2.1±1.2	2.5±1.3

Exercise as a purposive activity was seen in a small percentage of the study population (12.7%). An essentially similar percentage of participants from varying pain intensity performed regular exercise therefore, statistical significance was not found. Among the other factors, association of pain intensity with anemia and absenteeism was highly significant (P=0.000). Absenteeism was more common in those with increasing severity of dysmenorrhea. The mean number of days of absence from schools and colleges for those with moderate to severe dysmenorrhea was 2.21±1.2 days (not given in the table). An attempt was made to identify the mean work days lost per person experiencing dysmenorrhea with varying intensities. Absenteeism was found linearly associated to the severity of dysmenorrhea. Females experiencing mild pain on an average absented for one and half day a month while 2.1±1.2 and 2.5±1.3 days for those who experienced moderate and severe forms of dysmenorrhea respectively. However, the absolute number of days of absenteeism ranged from half a day to 3 days. Evidently the panorama of dysmenorrhea with its menstrual characteristics affects working ability of the females. An extremely significant association was found between the severity of dysmenorrhea and limitation in working capacity of females. An inverse relationship is exhibited between severity of dysmenorrhea and percentage of females reporting unaffected working ability while a proportional increase can be seen in percentage of females reporting clear inhibition of working ability.

Pain characteristics were also investigated in dysmenorrheic females. Location of pain is considered as an important criterion. Pain characteristics show some association with pain intensity in the selected subjects. It is evident from Table 5 there was an association between pain location and severity of pain. The Chi-square test shows extremely significant associations between intensity of pain and frequency of occurrence of pain as well as the pain length. (P=0.000). It can be seen from Table 5 that abdominal pain was most frequent symptom in females experiencing dysmenorrhea regardless of its intensity. Other locations for pain reported by the participants were either sides of abdomen, low back pain and pain in more than one region. Our results point out to certain pattern of pain occurrence, the percentage of females with mild and moderate dysmenorrhea reported higher frequency for lower abdomen and low back pain as against those with severe dysmenorrhea. Pain in more than one location was reported by women with severe dysmenorrhea in significantly higher percentage. It could be possible that in severe dysmenorrhea the pain intensity being high a perception of defused pain in the entire region of abdomen and back may occur.

**Table 5:** Distribution of pain characteristics according to dysmenorrhea intensity

Pain characteristics		Menstrual pain intensity			Total	Chi Sq

		Mild n (%)	Moderate n (%)	Severe n (%)		
**Pain Location**	Lower abdomen	83(50.3)	159(53.4)	70(46.4)	312(50.8)	25.3**
	Low back	46(27.9)	48(16.1)	19(12.6)	113(18.4)	
	sides of abdomen	12(7.2)	21(7.0)	14(9.3)	47(7.7)	
	Pain at different locations	24(14.6)	70(23.5)	48(31.8)	142(23.1)	
**Pain Characters**	Twisting	77(70.0)	139(62.6)	71(57.7)	287(63.1)	9.36^Ns^
	Sharp	20(18.2)	43(19.4)	28(22.8)	91(20.0)	
	Stinging	13(11.8)	34(15.3)	17(13.8)	64(14.1)	
	Mix	0(0.0)	6(2.7)	7(5.7)	13(2.8)	
**First experience of menstrual pain**	From the first period	78(49.4)	178(58.8)	80(54.4)	336(55.2)	4.03^Ns^
	After one year	46(29.1)	68(22.4)	36(24.5)	150(24.7)	
	Other	34(21.5)	57(18.8)	31(21.1)	122(20.1)	
**Frequency of occurrence pain**	More frequently (every periods)	90(44.5)	237(74.3)	131(87.3)	458(68.2)	87.46^***^
	Less frequently (Once in 3 month)	64(31.7)	58(18.2)	12(8.0)	134(20.0)	
	Rarely(Once in 6 months)	48(23.8)	24(7.5)	7(4.7)	79(11.8)	
**Initiation of pain**	Previous day	47(23.6)	76(23.2)	36(23.7)	159(23.4)	9.76^Ns^
	First day	98(49.2)	180(55.0)	86(56.6)	364(53.7)	
	Second day	48(24.1)	64(19.6)	24(15.8)	136(20.1)	
	Later	4(2.0)	4(1.2)	1(0.7)	9(1.3)	
	No fixed pattern	2(1.0)	3(0.9)	5(3.3)	10(1.5)	
**Pain length**	One day	76(38.0)	100(30.6)	23(15.1)	199(29.3)	27.44^***^
	1-2 days	82(41.0)	128(39.1)	68(44.7)	278(40.9)	
	2-3 days	33(16.5)	79(24.2)	47(30.9)	159(23.4)	
	Entire of period	9(4.5)	20(6.1)	14(9.2)	43(6.4)	

The descriptive terms used to characterize pain were twisting, sharp and stinging. All the dysmenorrheic females regardless of the severity reported essentially similar pain characteristics. The pattern of occurrence of pain was evident since a majority of females reported to experience twisting pain followed by sharp pain. Other information such as first experience of menstrual pain, frequency of pain occurrence, initiation and length of pain is also presented in the table. Considering the highest level of occurrence among the pain characteristics such as” experience of pain from the first period”, “occurrence of pain every period”, “initiation of pain from previous or first day” or” pain length” as the severe condition, it is obvious that severity of each variable mentioned, appeared with females having severe dysmenorrhea. Significantly higher percentage of girls with severe dysmenorrhea experienced pain every cycle, initiation of pain was reported from the first day and pain length was also long. This is a clear evidence of the sufferings among dysmenorrheic females which is directly proportional with mild, moderate and severe dysmenorrhea. We found extremely significant association between frequency of pain occurrence and dysmenorrhea as well as pain length and dysmenorrhea.

The most common symptom present in both dysmenorrheic and non dysmenorrheic girls during the menstrual periods was tiredness (56.8 and 37.8% respectively) and second most prevalent symptom was back pain (40.1% and 17.6%).

The information provided in Table 6 is interesting since the differences in the occurrence rate of certain discomforts among dysmenorrheic and non dysmenorrheic females indicates the extent of sufferings the dysmenorrheic females undergo with each cycle of menstruation. Also, the table provides the common problems of females regardless of menstrual pain. The uterine contraction that occurs during menstruation is painful is generally accompanied with other symptoms, including tiredness, painful/tender breasts, supra pubic cramping, backache, general pain and vomiting.

**Table 6:** Distribution of the subjects according to the menstrual symptoms and occurrence of dysmenorrhea

Menstrual symptoms	Groups		

	Dysmenorrheic	Non dysmenorrheic	Chi sq
**Tiredness**	397(56.8)	112(37.8)	30.15^***^
**Back pain**	280(40.1)	52(17.6)	47.7^***^
**Anger/short tempered/irritable**	185(26.5)	43(14.5)	16.86^***^
**Mood swings**	122(17.5)	28(9.5)	10.43**
**General aches and pains**	119(17.0)	15(5.0)	25.42^***^
**Acne**	105(15.0)	34(11.5)	2.13^Ns^
**Headaches**	85(12.2)	31(10.5)	0.586^Ns^
**Insomnia**	63(9.0)	10(3.4)	9.74**
**Vomiting**	57(8.1)	7(2.4)	11.61^***^
**Cramps**	56(8.0)	2(0.6)	20.42^***^
**Painful/tender breasts**	55(7.9)	5(1.7)	14.04^***^
**Altered appetite**	61(8.7)	10(3.4)	9.00**
**Less frequent symptoms**		
**Nausea, diarrhea swelling, craving**	2.8-4.8	0.6-3.4

Management of pain adopted by the selected girls is also presented in Table 7. It is obvious that a small proportion of girls reported to have sought medical advice, nevertheless an insignificant percentage of girls used self selected medicine without doctor’s consultation. Majority of dysmenorrheic females practiced staying in bed, having hot water bath, use of special food or drink to reduce pain and distraction by watching TV, reading etc. as a measure to relieve pain. As it can be seen from the table the most prevalent method was staying in bed.

**Table 7:** Menstrual pain management behaviors of the selected females

Menstrual pain intensity N=698	Menstrual pain management (%)						

	Pharmacological supplements		Non pharmacological Methods				

	over-the-counter medication	On doctors consultation	Stay in bed	Hot bath/use of heating pad	Distraction	Special food or drink*	Exercise
**Mild**	15(19.7)	31(17.4)	121(25.4)	63(28.5)	45(26.0)	32(30.2)	9(39.1)
**Moderate**	36(47.4)	74(41.6)	236(49.6)	107(48.4)	84(48.6)	47(44.3)	6(26.1)
**Severe**	25(32.9)	73(41.0)	119(25.0)	51(23.1)	44(25.4)	27(25.5)	8(34.8)
**Total**	76(6.1)	178(14.2)	476(38.0)	221(17.6)	173(13.8)	106(8.5)	23(1.8)

* considered to relieve sufferings.

## 4. Discussion

We found 70.2% of females suffering from primary dysmenorrhea in selected population. Our observation is consistent with previous studies reporting dysmenorrhea occurrence between 45 and 85% from India and other countries (Loto, 2008; Okusanya, 2009; Agarwal & Agarwal, 2010; Alaettin, 2010; Yasir, 2014). It is evident from results that nearly 70% of dysmenorrheic females experienced moderate to severe dysmenorrhea. While comparing prevalence of dysmenorrhea from other countries, Ethiopia reported 70% dysmenorrhea wherein 28.5% had moderate to severe, Malaysia reported an incidence of 6.8% having moderate to severe pain and Jordan reported 55.8% of the subjects had moderate to severe pain. One of the reasons for such vast differences could be due to the scales used for assessment (Lee, 2006; Zegeye, 2009; Al-Jefout, 2014).

In present study 20 and 11.8% dysmenorrheic girls reported having pain frequently and rarely (Table 2). The distribution pattern of pain frequency reported by the study population was found to be similar to the report from Hong Kong (Symphorosa, 2009). A study from India indicated 34% females experience dysmenorrhea more frequently (every month) (Agarwal & Agarwal, 2010). In comparison to this study, we found a markedly higher occurrence (68.3%) of frequent dysmenorrhea. It is worthwhile to highlight that female with more frequent and less frequent experiences of dysmenorrhea was found to be 88% and this proportion is considerably higher.

We found approximately 50% of dysmenorrheic girls remained absent from schools or colleges. In several studies of young women, rates of absenteeism ranged from 24 to 50% (Banikarim, 2000; Chia et al., 2013). Studies have demonstrated that menstrual pain restricts the movement and usual activity pattern of the females. It could also be argued that the perception of uneasiness and discomfort experienced during menstruation probably limits the movements more than the experience of pain.

Results exhibited 69.7% of subjects were moderately affected and 7.6% of both dysmenorrheic and non dysmenorrheic mentioned to have a clear inhibition of their activities. Other studies also reported such activity restriction (Tariq, 2009; Al-Asadi, 2013).

Interestingly we found higher percentage of Muslim girls’ experienced severe pain. A report has shown, that Muslim adolescents exhibited higher degrees of somatization, which may suggest that the cultural factor had a unique effect on the menstrual experiences (Goldestein-Ferber, 2006).

In only 12.7% of the study population exercise was a purposive activity. However, it is hypothesized that exercise works by improving blood flow at the pelvic level as well as stimulating the release of endorphins, which act as non-specific analgesics (Proctor & Farquhar, 2006). A recent review showed that in studies where more than 500 participants were included there tended to be no association between dysmenorrhea and exercise/physical activity patterns but smaller studies (<500 participants) reported positive associations, where bias is also more likely to be present (Daley, 2008).

We found tiredness and back pain as the most prevalent menstrual symptoms and our observations were similar to that reported by others (Agarwal & Agarwal, 2010; Aktaş, 2015).

It is worthwhile to comment that, despite the sufferings, only a small proportion of girls in our study had sought pharmacological management (25.5%) and 83.2% depended on non pharmacological methods. Only 14.2% had sought medical advice and this suboptimal use of the medical advice has also been reported by others (Banikarim, 2000; Lee, 2006; Konapur, 2014; Aktaş, 2015; Farotimi et al., 2015). The barriers to seek medical attention by dysmenorrheic females need exploration. It is important that health education on puberty and menstruation is regarded as inadequate for many young females in India and other nations. This ignorance might adversely affect the medical attention seeking behavior of dysmenorrheic females.
